# The dynamic impact of Joint Awareness on Quality of Life after Total Knee Arthroplasty: a longitudinal study

**DOI:** 10.1186/s13018-022-03456-z

**Published:** 2022-12-26

**Authors:** Qi Li, Qingqing Su, Yaoyao Zhang, Jing LYu, Yake Li, Haiyan Li

**Affiliations:** 1 School of Nursing, Qingdao University, Qingdao, Shandong China; 2 Department of Oral and Maxillofacial Surgery, Affiliated Hospital of Qingdao University, Qingdao, Shandong China; 3 Department of Joint Surgery, Affiliated Hospital of Qingdao University, 59 Hai’er Road, Qingdao, Shandong China

**Keywords:** Total Knee Arthroplasty, Joint Awareness, Quality of Life, Forgotten Joint Score-12, Longitudinal study

## Abstract

**Background:**

Joint Awareness is thought to be closely linked to Quality of Life (QoL) for patients undergoing Total Knee Arthroplasty (TKA), yet to date there have been no longitudinal studies to explore how Joint Awareness actually affects QoL. The purpose of this study was therefore to examine the development of Joint Awareness and QoL after TKA as well as the dynamic impact of Joint Awareness on QoL.

**Methods:**

A total of 342 patients were followed up at 3 months (T1), 6 months (T2), and 12 months (T3) after TKA. Joint Awareness was evaluated using the Forgotten Joint Score-12 (FJS-12), and QoL was measured by SF-36. We used repeated measures analysis of variance to estimate the development of Joint Awareness and QoL and employed a cross-lagged model to examine the dynamic relationship between Joint Awareness and QoL.

**Results:**

Both Joint Awareness and QoL improved with postoperative time (*p* < 0.001). Importantly, T1 Joint Awareness positively predicted T2 physical QoL (*p* < 0.001), and T2 Joint Awareness positively predicted T3 physical QoL (*p* < 0.001). Nevertheless, Joint Awareness had no predictive effect on mental QoL (*p* = 0.082–0.931).

**Conclusions:**

In different periods after TKA, Joint Awareness and QoL both increased monotonically, and Joint Awareness positively predicted physical QoL. These findings indicate that focusing on Joint Awareness may be a priority when trying to improve the postoperative life of patients.

## Background

Researchers have estimated that over 250 million patients are affected by arthritis around the world, and knee osteoarthritis (KOA) is the type of arthritis that is most associated with joint degeneration, pain, and disability [1]. For those with advanced KOA, Total Knee Arthroplasty (TKA) has become an effective treatment, and the volume of this surgery is expected to grow steadily worldwide over the next decade [2, 3]. After undergoing TKA, patients usually become aware of pain relief, recovered knee functionality, and overall Quality of life (QoL) improvements [4], and the treatment outcomes of TKA can also be objectively evaluated through image-based assessments, the Knee Society Score (KSS), and implant survivorship [5].

In recent years, joint surgeons have begun to place more emphasis on the views of patients, in addition to these objective evaluations [6]. Joint Awareness is a new form used for patient-reported outcomes (PRO) and refers to a state where a patient is able to forget they have a joint prosthesis completely during all daily activities [7, 8]. Among TKA patients who recuperated well, only 66.1% had a high degree of Joint Awareness [9]. Notably, Joint Awareness has been closely linked to QoL for patients undergoing TKA, and that the correlation with physical function and pain has been found to be higher than that of psychological state [10]. Joint Awareness and QoL are necessarily dynamic concepts, but unfortunately there are only cross-sectional studies on the two together; no longitudinal study that explores how Joint Awareness affects QoL after TKA yet exists [10, 11]. Therefore, for this study, we implemented a longitudinal design aimed at identifying the development of Joint Awareness and QoL after TKA and analyzing the dynamic impact of Joint Awareness on QoL in order to provide a reference to help improve the quality of postoperative life of patients after TKA.

## Methods

### Participants

This longitudinal study was carried out in a tertiary hospital in Qingdao, China. Only patients who had undergone a cemented posterior stabilized TKA (ZUK; Zimmer, Warsaw, IN) were recruited. The inclusion criteria were as follows: (1) Patients must have undergone TKA for knee osteoarthritis. (2) Patients must have had complete postoperative data. Likewise, the exclusion criteria were as follows: (1) Patients who had undergone bilateral or revision TKA. (2) Patients who had been treated with any other surgery on the lower extremities. (3) Patients who experienced complications, such as prosthetic loosening or deep vein thrombosis.

The sample size was estimated based on a calculation of 5–10 times the number of scale entries [12]. There were 48 items measured in our study. We therefore expected that at least 300 participants would be required, considering a sample dispersion rate of 20%. All operations were performed by joint surgeons in the same medical group, and the patients received unified and standardized postoperative rehabilitation after TKA in our hospital. Patient demographic data were collected prior to TKA, and we recorded the degree of Joint Awareness and QoL at 3 months (T1), 6 months (T2), and 12 months (T3) after TKA.

### Ethical considerations

The study was approved by the ethical committee of the Affiliated Hospital of Qingdao University, and all participants were informed about the aims of the study and volunteered to participate.

### Measurements

#### Demographic characteristics

As mentioned above, demographic data included gender, age, education level, surgical site, BMI, ASA class and postoperative analgesia type (Table 1).

**Table 1:** Demographic characteristics

Variable	Included (*n* = 342)	Excluded (*n* = 58)	*p* value
Sex (*n*, %)			0.477
Female	218 (63.7)	40 (69.0)	
Male	124 (36.3)	18 (31.0)	
Age, years (*n*, %)			0.008
≤ 60	111 (32.5)	13 (22.4)	
> 60	231 (67.5)	45 (77.6)	
Basic education (*n*, %)			0.307
Secondary/comprehensive school (or less)	267 (78.1)	44 (75.9)	
Senior high school	52 (15.2)	9 (15.5)	
Junior college or above	23 (6.7)	5 (8.6)	
Side (*n*, %)			0.764
Right	166 (48.5)	27 (46.6)	
Left	176 (51.5)	31 (53.4)	
BMI (kg/m^2^ ± SD)	27.02 ± 2.20	27.19 ± 2.14	0.100
ASA class (*n*, %)			0.813
II	160 (46.8)	28 (48.3)	
III	182 (53.2)	30 (51.7)	
Postoperative analgesia type (*n*, %)			0.386
Conventional analgesic	39 (11.4)	7 (12.1)	
PCIA	303 (88.6)	51 (87.9)	

BMI, Body Mass Index; ASA, American Standards Association; PCIA, Patient Controlled Intravenous Analgesia

#### The Forgotten Joint Score-12 (FJS-12)

The FJS-12 was developed by Behrend in 2012 to reflect the degree to which artificial joints have become forgotten in daily life [6]. The scale consists of 12 questions, and all questions are answered with never, almost never, seldom, sometimes, mostly, or “not relevant to me”, corresponding to 0 to 4 points and a missing value, respectively. Higher scores on the FJS-12 mean patients are more likely to have forgotten that they have TKA. The English FJS-12 has been shown to be reliable and effective and has also been translated into many languages. The Cronbach’s *α* of the Chinese version is 0.907 [10].

#### The 36-Item Medical Outcomes Study Short-Form Health Survey (SF-36)

We used the SF-36 to investigate the QoL of the patients in this study. This survey was developed by the US Medical Outcomes Study Group and consists of two portions, the Physical Component Summary (PCS) and the Mental Component Summary (MCS). The scale itself uses a percentage system, and the scores of factor are converted to a standard score of 0–100 as well. The scores of PCS subscales and MCS subscales are calculated separately. Higher scores indicate a higher QoL, and the retest reliability of the survey is 0.7 to 0.9 [13].

### Data analysis

All data analysis was conducted in SPSS software version 26.0 or Mplus statistical software version 8.3. Descriptive statistics were used to determine the distributions of demographic characteristics and the scores of FJS-12 and SF-36. Continuous variables were expressed as means and standard deviations and categorical variables as absolute values and percentages. We used repeated measures analysis of variance [14] to analyze both the development of Joint Awareness and QoL. Cross-lagged analysis [15] was employed to examine the temporal order and overall causal direction of Joint Awareness and QoL. Model fit was evaluated by chi-squared statistic, Comparative Fit Index (CFI), Tucker-Lewis index (TLI), Root Mean Square Error of Approximation (RMSEA), and Standardized Root Mean Square Residual (SRMR) [16], where CFI > 0.95, TLI > 0.90, RMSEA < 0.10, and SRMR ≤ 0.08 indicate a normal fit for each measure [17]. We considered a *p* value less than 0.05 to indicate a statistically significant test result.

## Results

### Patient Characteristics

A total of 400 participants enrolled in this study, but 58 of them were excluded due to incomplete data. As a result, 342 patients (85.5%) were included in the final analysis. Demographic comparisons between patients who were included in the present study and those who were excluded are shown in Table 1. Among the included patients, 63.7% were women; their BMI was 27.02 ± 2.20 kg/m^2^; 53.2% were ASA class III; and 88.6% had a postoperative analgesia type of PCIA. The included patients were younger than the excluded group (32.5% vs. 22.4%, *p* = 0.008), though the available demographics were comparable between the 2 groups.

### Scores of Joint Awareness and QoL at different times

At T1, the score for FJS was 29.43 ± 8.93, the score for PCS was 42.30 ± 9.57, and the score for MCS was 57.39 ± 7.90. Next, at T2, the score was 44.51 ± 9.52 for FJS, 51.61 ± 9.66 for PCS, and 58.30 ± 7.66 for MCS. Finally, at T3, the scores for FJS, PCS, and MCS were 55.73 ± 9.45, 58.42 ± 10.15, and 61.05 ± 7.69, respectively.

### Development of Joint Awareness and QoL

**Fig. 1:**
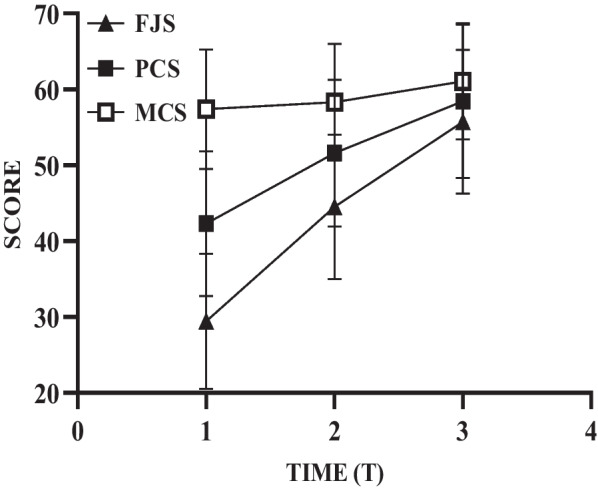
The growth of Joint Awareness and QoL. QoL, quality of life; FJS, Forgotten Joint Score; PCS, Physical Component Summary; MCS, Mental Component Summary; Time 1, 3-month follow-up; Time 2, 6-month follow-up; Time 3, 12-month follow-up

The results of repeated measures analysis of variance showed that the time effect of Joint Awareness was statistically significant (*F* = 4101.784, *p* < 0.001), indicating that Joint Awareness changed over time. More specifically, Table 2 shows that Joint Awareness improved more from T1 to T2 than from T2 to T3 (difference 15.086 vs. 11.217, *p* < 0.001). We also found that the time effect of physical and mental QoL was statistically significant (*F* = 1464.363, *p* < 0.001; *F* = 132.792, *p* < 0.001), indicating that QoL changed over time as well. Here, physical QoL improved more from T1 to T2 than from T2 to T3 (difference 9.308 vs. 6.814, *p* < 0.001), yet mental QoL improved less from T1 to T2 than from T2 to T3 (difference 0.918 vs. 2.742, *p* < 0.001) (Table 2). Furthermore, Table 2 also shows that the growth rate of mental QoL was much lower than that of physical QoL from T1 to T3 (difference 3.660 vs. 16.121, *p* < 0.001). In addition, Fig. 1 shows the growth of FJS, PCS, and MCS after TKA, and we can also see that the level of Joint Awareness and QoL increased with time.

**Table 2:** Development of Joint Awareness and QoL by repeated measures analysis of variance

Variable	Time (A, B)	Difference (B − A)	SE	95% CI		*p* value
				Lower	Upper	
FJS	T1, T2	15.086	0.267	14.561	15.610	< 0.001
	T2, T3	11.217	0.256	10.714	11.720	< 0.001
	T1, T3	26.303	0.290	25.732	26.874	< 0.001
PCS	T1, T2	9.308	0.206	8.902	9.714	< 0.001
	T2, T3	6.814	0.260	6.303	7.325	< 0.001
	T1, T3	16.121	0.318	15.496	16.747	< 0.001
MCS	T1, T2	0.918	0.110	0.701	1.134	< 0.001
	T2, T3	2.742	0.187	2.374	3.111	< 0.001
	T1, T3	3.660	0.225	3.218	4.102	< 0.001

QoL, Quality of Life; FJS, Forgotten Joint Score; PCS, Physical Component Summary; MCS, Mental Component Summary; SE, Standard Error; CI, Confidence Interval; T1, Time 1 (3-month follow-up); T2, Time 2 (6-month follow-up); T3, Time 3 (12-month follow-up)

### The dynamic relationship between Joint Awareness and QoL

**Fig. 2:**
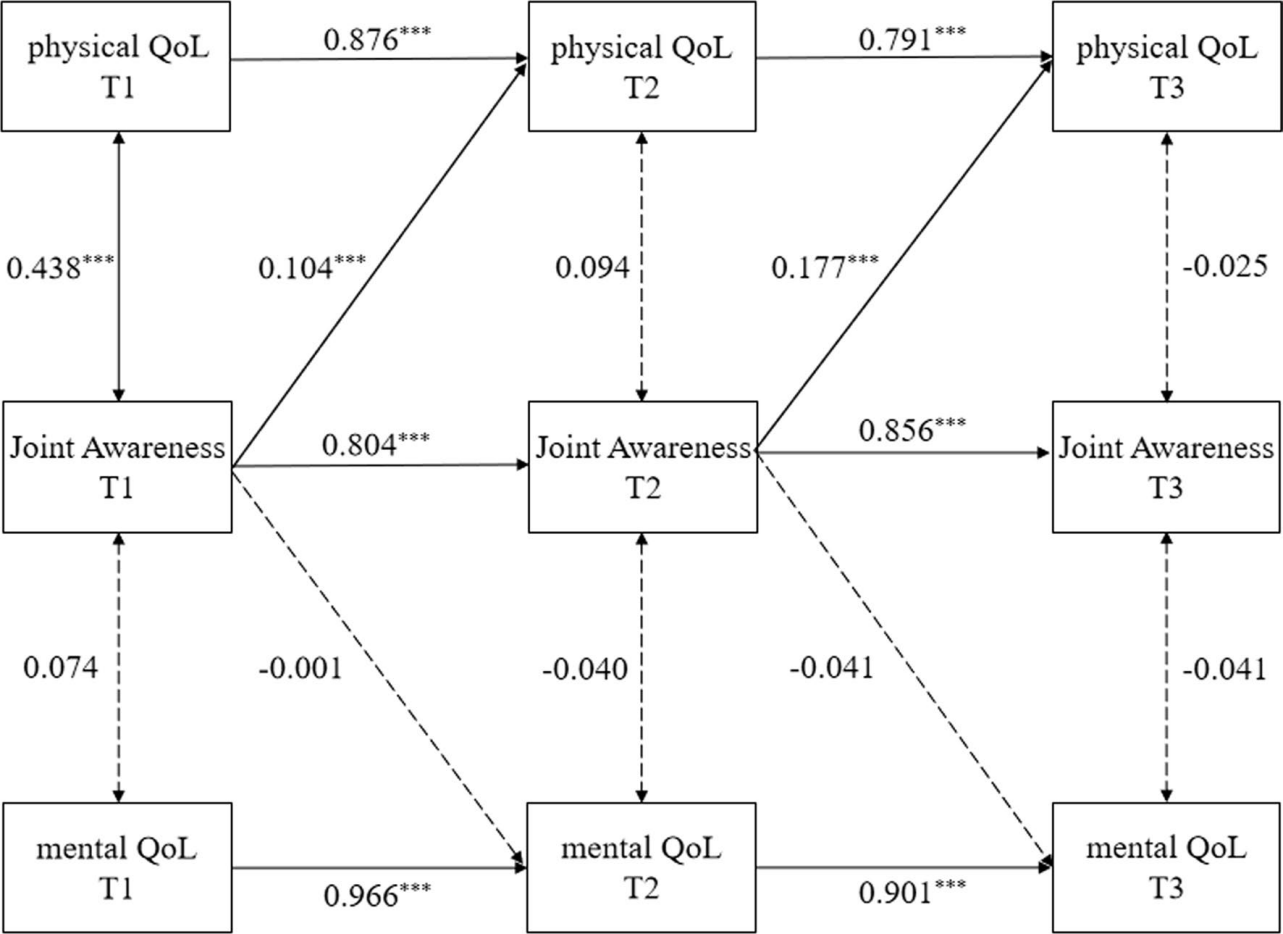
The dynamic relationship between Joint Awareness and QoL. The figures represent the standardized regression coefficients of significant paths; Significant paths are marked in solid lines and insignificant paths are marked in dashed lines; ***p < 0.001; QoL, quality of life; T1, Time 1 (3-month follow-up); T2, Time 2 (6-month follow-up); T3, Time 3 (12-month follow-up)

The cross-lagged model was generated as displayed in Fig. 2, and the summary statistics show that the model fit was acceptable: χ^2^ = 53.972, df = 14, *P* < 0.001, RMSEA = 0.091, CFI = 0.989, TLI = 0.975, SRMR = 0.019. The auto-regressive effects for Joint Awareness, physical QoL and mental QoL were statistically significant (*β* = 0.804–0.856, *p* < 0.001; *β* = 0.791–0.876, *p* < 0.001; *β* = 0.901–0.966, *p* < 0.001), and there was a reciprocal relation between Joint Awareness and physical QoL with positive cross-lagged effects observed from T1 Joint Awareness to T2 physical QoL (*β* = 0.104, *p* < 0.001), as well as from T2 Joint Awareness to T3 physical QoL (*β* = 0.177, *p* < 0.001). This means that T1 Joint Awareness was a positive predictor of T2 physical QoL, and T2 Joint Awareness was a positive predictor of T3 physical QoL. Nevertheless, the cross-lagged model revealed no statistically significant cross-lagged pathway from T1 Joint Awareness to T2 mental QoL (*p* = 0.931) or from T2 Joint Awareness to T3 mental QoL (*p* = 0.082). There was no significant predictive effect of Joint Awareness on mental QoL.

## Discussion

As far as we know, this is the first longitudinal study to examine the dynamic impact of Joint Awareness on QoL after TKA. We found that Joint Awareness and QoL both improved over time. Joint Awareness at 3 months had a positive predictive effect on physical QoL at 6 months, and Joint Awareness at 6 months also predicted positively physical QoL at 12 months.

An important expectation of TKA patients was forgetting the artificial joint in daily life [6], and we found that this was indeed the case in our sample as postoperative time increased. In fact, our research is similar to the existing studies that have shown that the postoperative awareness of a joint gradually decreases for patients over time [18, 19]. The reason for this may be that changes in surgical implementation of knee prostheses have occurred and now-standardized rehabilitation has enhanced the degree of adaptation to prostheses [20]. Furthermore, the growth from 3 to 6 months was higher than that from 6 to 12 months after TKA. This result is similar to the study of Hiyama, which showed that the changes in forgotten joint were mostly observed within the first 6 months and that patients reached a plateau in their the sense of knee prosthesis at 6 to 12 months [21].

Nevertheless, there were almost no TKA patients who had completely forgotten the replacement knee in our sample. A FJS-12 score higher than 84.38 indicates that a patient has forgotten about the joint [22]. None of the patients investigated in our study reach this state, which suggests that patients need more than one year to adapt to joint replacement. Improving the degree of forgotten joint is the target index for postoperative satisfaction of TKA, and scholars have suggested that Joint Awareness should be routinely followed up after TKA [23, 24]. However, current systems for evaluating the postoperative efficacy often focus on objective evaluation, possibly ignoring the subjective feelings of patients [25]. Therefore, we suggest that the degree of forgotten knee should be taken into account. Exploring programs to manage the degree of forgotten knee is a direction for new research.

We also found that QoL advanced over time, consistent with other studies that have found that QoL at 3 months after TKA was better compared to that before TKA and that people resumed the vast majority of their daily activities at 12 months after TKA [9, 26]. For a patient to undergo TKA, it must be perceived as high value in improving knee functions and relieving pain, and postoperative high intensity exercise and remote rehabilitation may be helpful in reducing joint stiffness [27, 28]. Therefore, the physiological QoL can be improved accordingly during postoperative time. In order to maximize the benefits of TKA, rehabilitation training should be carried out as early as possible, and the guidance of functional exercise after discharge should also be prioritized [29]. In addition, our study showed that the change in mental QoL is less than that in physical QoL. This is also consistent with the study of Xie, which had shown that PCS was better than MCS and that overall MCS remained stagnant at 6 months after surgery [30]. This is not difficult to explain when considering that mental health is affected by personality characteristics, interpersonal communications, and other factors besides physical conditions. Furthermore, psychological status is also closely related to pain and knee function recovery [31]. These two aspects provide a necessary reference for clinicians to focus more on preoperative communication and postoperative psychological support, possibly even helping them to find ways to reduce unrealistic patient expectations [32].

Joint Awareness positively predicted physical QoL at the successive time points. Enhancing the degree of forgotten knee prostheses may also be helpful in improvement postoperative physical function after TKA. The potential reasons for this finding may be that Joint Awareness of patients may become more stable over time, and the unnatural feeling of the prosthesis may decrease. The presence of the prosthesis may also become less visible in activities, which may subtly promote the carrying out of physical functions. There are similar findings from one cross-sectional study [10] that shows that Joint Awareness is highly correlated with QoL on the physical subscale. Nonetheless, there is no existing study that explores the dynamic relationship between Joint awareness and QoL; therefore, we are unable to compare our result directly with other conclusions. It seems intuitively meaningful to predict QoL of patients, using Joint Awareness since this may help patients improve their prognoses. However, Joint Awareness had no predictive effect on mental QoL. Our results are consistent with the study of Cao, which found that FJS was weakly positively correlated to MCS [10]. Although it may not be surprising, positive emotions are beneficial to the recovery of joints, and mental health is affected by many factors, such as self-efficacy and cognition [33, 34]. As a result, future research should explore the dynamic relationship between forgotten joint and psychological QoL so as to increase patient awareness of the full spectrum of diseases and treatment methods for patients.

Information about the development of Joint Awareness and QoL and how Joint Awareness can predict future physical QoL may be useful in individualizing rehabilitation plans. On this basis, our study can pave the way toward developing targeted interventions to help patients to obtain a reasonable cognition of Joint Awareness and to help sequentially improve patient’s postoperative living standards. However, we must also note several limitations of this study. The first is that our study did not track Joint Awareness on QoL longer than one year after TKA. Future research can extend the follow-up time. Second, our study was conducted in only one hospital in Qingdao, China, and the number of patients was relatively limited, which may have led to the bias in the results. Subsequent studies should increase the sample size and the number of centers included in order to test the relationship between Joint Awareness on QoL more accurately. Finally, only subjective measurements were recorded in this study. Lower-extremity-specific performance and string-specific physical function can also be quantitatively tested in the future.

## Conclusions

Overall, we found that Joint Awareness and QoL both improved after TKA and that Joint Awareness positively predicts physical QoL over time. To our knowledge, our study is one of the only studies to have used a longitudinal design to evaluate the impact of Joint Awareness on QoL. These results may contribute to a better understanding of the changes of Joint Awareness and QOL after TKA.

## Data Availability

The datasets used and/or analyzed during the current study are available from the corresponding author on reasonable request.
